# Association between circulating irisin level and depression: a systematic review and meta-analysis

**DOI:** 10.1080/07853890.2025.2521424

**Published:** 2025-06-20

**Authors:** Chengyan Han, Zining Zhou, Jianxing Zhao, Zhouli Shao, Peng Sun

**Affiliations:** a School of Rehabilitation, Hangzhou Medical College, Hangzhou, Zhejiang, China; b Center for Rehabilitation Medicine, Rehabilitation & Sports Medicine Research Institute of Zhejiang Province, Department of Rehabilitation Medicine, Zhejiang Provincial People’s Hospital (Affiliated People’s Hospital,), Hangzhou Medical College, Hangzhou, Zhejiang, China

**Keywords:** Depression, irisin, systematic review, meta-analysis

## Abstract

**Background:**

Previous studies have presented controversial results about the association between irisin and depression. This research is designed to explore the circulating irisin levels in depressive patients.

**Methods:**

From the earliest available records up to 18 July 2024, searches were conducted in databases of PubMed, Embase, Web of Science, the Cochrane Library, and Scopus, in order to identify the relevant articles assessing the correlation between irisin and depression in humans. Two reviewers screened reports, retrieved and collected data independently.

**Results:**

Totally, eight articles with 2044 samples (733 depressive patients and 1311 non-depressive individuals) were involved. The results indicated lower circulating irisin levels in depression (standardized mean difference [SMD] = 0.60, 95% confidence interval [CI]: 0.08 to 1.12, *p* = 0.02). Subgroup analysis revealed decreased irisin levels in depressive Asian patients (SMD = 0.47, 95% CI: 0.05 to 0.90, *p* = 0.03), sample size greater than 100 participants (SMD = 1.20, 95% CI: 0.48 to 1.93, *p* = 0.001), ELISA kits not from Phoenix Pharmaceuticals (SMD = 0.47, 95% CI: 0.05 to 0.90, *p* = 0.03), blood sample of serum (SMD = 0.79, 95% CI: 0.18 to 1.40, *p* = 0.01), studies with two genders (SMD = 0.85, 95% CI: 0.26 to 1.43, *p* = 0.005). Moreover, six studies computed the correlation factor (r) values between irisin levels and depressive scores, and the combined findings indicated an inverse correlation for these two variables (r = −0.47, 95% CI: −0.69 to −0.24, *p* < 0.001).

**Conclusions:**

The results of this research demonstrated that irisin levels were decreased in depressive patients, and there was a negative correlation between irisin levels and depressive scores, indicating that the lower the circulating irisin level, the more severe the depressive symptoms.

## 1. Introduction

Depression is a common mental illness, with over 400 million people suffering from it all over the world [1]. Depression is linked to mood dysregulation, and some patients may even experience self-mutilation, suicidal behaviour, and other psychiatric symptoms. The pathological mechanisms of depression are multifaceted [2]. Primarily, there is a dysregulation of monoamine neurotransmitters, including serotonin, norepinephrine, and dopamine, along with abnormalities in glutamatergic neurotransmitters, significantly impacting mood regulation and neural function. Besides, malfunctions in the neuroendocrine system, specifically in the hypothalamic–pituitary–adrenal (HPA) axis and the hypothalamic–pituitary–thyroid (HPT) axis, contribute to depressive symptoms. Regarding neural plasticity, the hippocampus atrophies due to various adverse factors, and the activities and connections of neurons in the prefrontal cortex change, leading to a decline in the brain’s emotional regulation ability. Moreover, in terms of neuroinflammation and immunity, an increase in inflammatory factors activates immune cells, inducing oxidative stress that damages nerve cells through processes like lipid peroxidation, protein oxidation, and DNA damage. Additionally, genetic factors significantly contribute to depression.

The diagnosis of depression is complex, and it requires the integration of information from multiple aspects. Generally, psychiatrists make judgments based on clinical interviews, symptom assessment tools such as the Hamilton Depression Rating Scale (HAMD), and the exclusion of other possible causes. In the treatment of depression, in addition to medication, non-pharmacological treatments such as exercise have also been proved to be effective [3]. However, due to the complexity of symptoms, the lack of objective diagnostic indicators, insufficient treatment compliance and other factors, the treatment of depression still faces certain difficulties. With a lot of unknown areas of pathogenesis, research on biochemical molecules related to depression remains a hot topic. At present, more and more studies focus on the functions of muscle factors in the identification and management of depression.

Irisin, as a new muscle factor, was first reported in the journal Nature in 2012 [4]. The production of irisin is mainly related to a series of physiological processes triggered by exercise. When the body is exercising, skeletal muscles will secrete irisin through the PGC- 1α/FNDC5/irisin signal pathway. Subsequently, irisin is released outside the cells and enters the bloodstream to exert its physiological effects [5,6]. The physiological functions of irisin are multiple, including modulating energy metabolism, improving circulatory function, exerting neuroprotective effects, and enhancing skeletal muscle quality [7,8].

Moreover, irisin has the ability to pass through the blood-brain barrier, thereby acting in alleviating neuroinflammation, regulating neuroplasticity, and resisting apoptosis [9]. Researchers have confirmed that irisin is also found in the brain, which is associated with neurological and psychiatric dysfunctions, such as depression [10,11]. Irisin is also expressed in the limbic system, especially in amygdala, thalamus and hippocampus [12]. The limbic system is considered to secrete neurotransmitters, like serotonin, dopamine, and norepinephrine, associated with the regulation of emotion.

Studies have demonstrated the relationship between irisin and depression. It is believed that the levels of irisin in depressive patients are lower than non-depressive individuals [13,14]. Similarly, irisin levels in cerebrospinal fluid are negatively correlated with the severity of depression [15]. On the other hand, animal studies have revealed that the FNDC5/irisin levels in the hippocampus of depressive model rats are reduced [16,17]. Furthermore, irisin may be involved in the treatment of depression by either injecting exogenous irisin or increasing endogenous irisin levels through exercise in depressive animal models with the improvement in behavioural tests such as forced swimming test, open field test, sugar water preference test, as well as tail suspension test [18,19]. However, some studies have suggested that the difference of irisin levels between depressive patients and healthy individuals is not significant [20].

Based on the controversies above, our study intended to clarify the connection between circulating irisin and depression, in order to provide new ideas for the prevention and treatment of this disease.

## 2. Materials and methods

Our research was carried out with reference to the Meta-analysis of Observational Studies in Epidemiology (MOOSE) guidelines and the Preferred Reporting Items for Systematic Reviews and Meta-Analyses (PRISMA) statement. In addition, registration of this study was completed in the international prospective register of systematic reviews (PROSPERO: CRD42024569648).

### 2.1. Search strategy

Articles of interest were searched in PubMed, Embase, Web of Science, the Cochrane library and Scopus, from inception dates to 18 July 2024 with all languages. The search terms were ‘irisin’, ‘FNDC5’, ‘fibronectin type III domain-containing protein 5 precursor’, ‘depression’, ‘depressive’, ‘dysthymia’, ‘mood disorder’ and ‘affective symptom’. The search strategy for detail is in Appendix 1. In addition, we searched the websites of some medical professional societies, the websites for clinical trial registration, databases for grey literature, and manually checked the reference lists of relevant articles to ensure the comprehensiveness of the included studies.

### 2.2. Selection criteria

The following were the inclusion criteria: (i) The type of research should be case-control study or cohort study on humans. (ii) The observation groups were depressive patients with the diagnoses of ICD/DSM criteria or depressive symptoms measured by standardized questionnaires. The control groups included people without depression. All participants were adults (aged 18 or older). (iii) The contents of research should be related to the association between circulating irisin level and depression.

The following criteria were applied for exclusion: (i) studies with case numbers less than ten in either arm; (ii) literature that was repeatedly published; (iii) literature with unavailable data; (iv) participants with pregnancy.

### 2.3. Outcome indicators

The primary outcomes of our meta-analysis were the irisin levels in peripheral blood of the depressive and the control groups. The second outcome of interest was the correlation between circulating irisin levels and depressive scores, including correlation coefficient (r) and 95% confidence interval (CI).

### 2.4. Data extraction

Study selection and data collection were accomplished *via* the individual work of two evaluators. Any discrepancies were discussed and decided by the research group. The following data were extracted by an Excel form: primary author, release year, country, number of participants enrolled, age, gender, coexisting diseases, scales used to evaluate depression, circulating irisin levels and the correlation factor linking irisin levels and depressive scores. For the studies with unavailable data, the first or corresponding authors were contacted if necessary.

### 2.5. Quality evaluation

Quality evaluation was conducted based on Newcastle–Ottawa Quality Assessment Scale (NOS) with three criteria: selection, comparability and exposure. The study was regarded as high quality if the rating was seven to nine points, moderate quality if the rating was five to six points and low-quality otherwise.

### 2.6. Statistical analysis

The statistics of the data were carried out with the help of RevMan 5.3 (Nordic Cochrane Centre, Copenhagen, Denmark) and Stata 16.0 (Stata Corp, College Station, TX, USA). Considering that the data of outcomes were continuous and the measurement standards of circulating irisin levels were varied, standardized mean difference (SMD) was adopted as the effect variable with 95% CI. Random effects models were selected in the case of high heterogeneity with *p* < 0.1 and I^2^ > 50% of Cochran’s Q test. Conversely, fixed effects models were used. Sensitivity analysis was performed to decide the influence of each report on the synthesized results.

Subgroup analysis was conducted by race, sample quantity, gender, ELISA kit, blood sample and coexisting diseases. The correlation factor was computed through the reciprocal of variance and Fisher’s Z transformation. Egger’s regression test was used for the analysis of publication bias. It was deemed to be statistically meaningful when the value of *P* was less than 0.05.

## 3. Results

### 3.1. Literature search results

**Figure 1.**
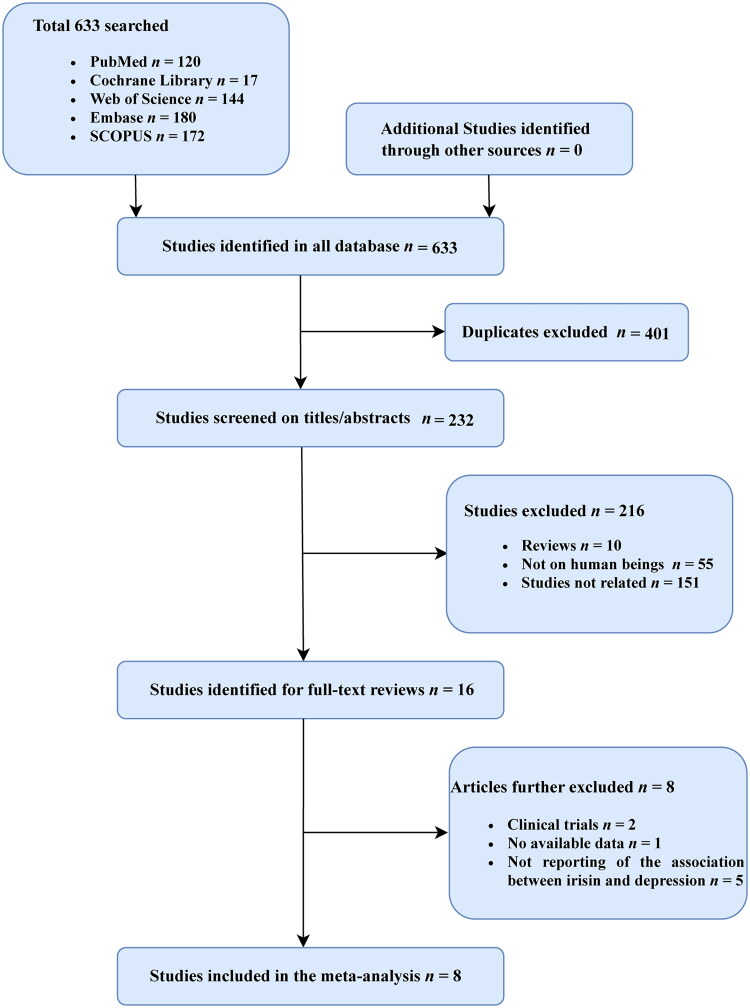
Flow chart of study selection process.

Totally, 633 studies were retrieved, including 120 in PubMed, 180 in Embase, 144 in Web of Science, 17 in the Cochrane library and 172 in Scopus. Among them, 401 studies were excluded due to duplication, 216 studies were excluded after reading the titles and abstracts and another eight studies were removed after reading the whole article. Apart from these databases, no additional studies satisfying the inclusion criteria were discovered on other websites. Eight articles, which were all case-control studies, were ultimately included [13–15,20–24] (Figure 1).

### 3.2. Study characteristics

There were a total of 2044 samples in all, involving 733 patients with depression and 1311 individuals without depression. These studies were conducted in six different countries (three from Turkey, one from Poland, two from China, one from Brazil, one from Germany). Among them, half had sample sizes greater than 100, while the rest were less than 100. All studies evaluated the circulating irisin levels, and the correlation coefficients of circulating irisin levels with depression scores were reported in six studies. To assess the depression scores, three studies used the Hamilton Rating Scale for Depression (HAM-D), two used the Geriatric Depression Scale (GDS), two used Patient Health Questionnaire 9 (PHQ-9), and the other one used the Beck Depression Inventory (BDI). In all studies, irisin levels were measured by intravenous blood after fasting and tested using enzyme-linked immunosorbent assay (ELISA), with kits purchased from seven companies, including Phoenix Pharmaceuticals (USA), Phoenix Pharmaceuticals (Germany), Dynex Technologies (USA), GUXI (China), Aviscera Biosciences (USA), SunRed (Turkey), Elabscience Biotechnology (China). Six participants suffered from other diseases, including coronary heart disease, obesity, fibroma, diabetes, stroke, and dementia. The study characteristics are abstracted in Table 1.

**Table 1.** Study characteristics.

		Sample size	Age		Sex ratio(m/f)				
Study	Country	Depression/Non-depression	depression	Non-depression	depression	Non-depression	Coexisting diseases	Depression scale	Outcomes
Gonçalves 2022 [15]	Brazil	25/36	70.8 ± 7.1	71.2 ± 6.0	9/16	15/21	dementia	GDS-15	①
Cicek 2023 [13]	Turkey	117/59	35.02 ± 12.93	34.64 ± 12.36	27/90	14/45	/	HAM-D	①②
Han 2019 [21]	China	53/156	59.58 ± 13.32	60.33 ± 10.74	30/23	86/70	coronary heart disease	PHQ-9	①②
Tu 2018 [14]	China	370/835	69 ± 15.56	64 ± 14.07	210/160	430/405	stroke	HAM-D	①②
Gorska 2023 [22]	Poland	57/132	72.9 ± 4.0	72.5 ± 4.9	11/46	50/82	diabetes mellitus	GDS-30	①②
Samanci 2019 [20]	Turkey	34/14	NA	NA	0/34	0/14	fibromyalgia syndrome	BDI	①
Hofmann 2016 [23]	Germany	49/49	43.7 ± 13.5	44.1 ± 11.7	0/49	0/49	Obesity	PHQ-9	①②
Erzin 2020 [24]	Turkey	30/28	40.4 ± 13.4	38.0 ± 9.4	12/18	8/20	/	HAM-D	①②

Notes: ①. Peripheral irisin levels of depression and non-depression; ②. Correlation coefficients of circulating irisin levels and depressive scores.

### 3.3. Methodological quality assessment

The NOS scores of the included ten studies were all six points or above (Table 2). Among them, six were high quality, while the remaining two were moderate quality. In terms of ‘Selection’, there were five articles without showing the consecutive or obviously representative series of cases and four articles without describing the history of depression of controls. Considering the domain of ‘Comparability’, four studies controlled only the most important confounding factors like age and gender with the omission of some additional factors such as BMI and the level of blood sugar. When it came to the turn of ‘Exposure’, three articles did not demonstrate whether the ascertainment of exposure was blind.

**Table 2.** Quality assessment by NOS of studies in meta-analysis.

Item/Study		Gonçalves 2022 [15]	Cicek 2023 [13]	Han 2019 [21]	Tu 2018 [14]	Gorska 2023 [22]	Samanci 2019 [20]	Hofmann 2016 [23]	Erzin 2020 [24]
Selection	1. Adequate definition of cases	*	*	*	*	*	*	*	*
	2. Representativeness of cases	*			*			*	
	3. Selection of controls	*	*	*	*	*	*	*	*
	4. Definition of controls		*		*	*			*
Comparability	Comparability of cases and controls	**	**	*	*	*	*	**	**
Exposure	1. Ascertainment of exposure	*		*	*	*	*		
	2. Same method of ascertainment for cases and controls	*	*	*	*	*	*	*	*
	3. Non-response rate	*	*	*	*	*	*	*	*
Total scores		8	7	6	8	7	6	7	7

### 3.4. Primary outcomes and subgroup analysis

**Figure 2.**
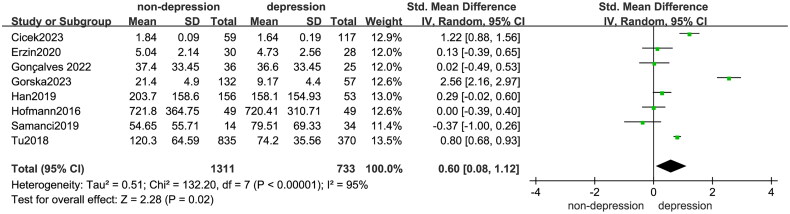
Forest Plot of meta-analysis with eight studies on circulating irisin levels in depression and non-depression.

Our primary outcomes were the irisin levels in depression and non-depression. The heterogeneity was significant among those studies (I^2^ = 95%, *p* < 0.1); therefore, random effects models were carried out. The synthesized results suggested that compared with the control groups, peripheral irisin levels in depressive patients were lower (SMD = 0.60, 95% CI: 0.08 to 1.12, *p* = 0.02) (Figure 2).

To further explore the influencing factors of outcomes, subgroup analyses were carried out by race, sample quantity, gender, ELISA kit, blood sample, and coexisting diseases (Table 3).

**Table 3.** Subgroup analyses of irisin levels in depression and non-depression.

		Heterogeneity		Meta-analysis		Heterogeneity between subgroup	
Subgroup	No. of studies	*I^2^*	*p* value	*SMD*(95%CI)	*p* value	*I^2^*	*p* value
Race							
Asian	5	88%	<0.001	0.47(0.05 to 0.90)	0.03	0%	0.67
Others	3	98%	<0.001	0.87(-0.86 to 2.59)	0.33		
Sample quantity							
≥100	4	96%	<0.001	1.20(0.48 to 1.93)	0.001	89.7%	0.002
< 100	4	0%	0.66	−0.02(-0.27 to 0.23)	0.87		
ELISA kits							
Phoenix Pharmaceuticals	3	98%	<0.001	0.87(-0.86 to 2.59)	0.33	0%	0.67
Others	5	88%	<0.001	0.47(0.05 to 0.90)	0.03		
Blood sample							
serum	6	95%	<0.001	0.79(0.18 to 1.40)	0.01	80.0%	0.03
plasma	2	0	0.95	0.01(-0.30 to 0.32)	0.94		
Gender							
female only	2	0	0.32	−0.1(-0.44 to 0.23)	0.55	86.9%	0.006
two genders	6	95%	<0.001	0.85(0.26 to 1.43)	0.005		
Coexisting diseases							
with other disease	6	96%	<0.001	0.57(-0.09 to 1.23)	0.09	0%	0.85
without other disease	2	92%	<0.001	0.69(-0.37 to 1.76)	0.20		

The subgroup analysis by race found that there was a decrease of irisin level in depressive groups compared with the control groups only in Asians (SMD = 0.47, 95% CI: 0.05 to 0.90, *p* = 0.03). When stratified by ELISA kits, there were significantly lower irisin levels in depressive patients with studies using ELISA kits not from Phoenix Pharmaceuticals (SMD = 0.47, 95% CI: 0.05 to 0.90, *p* = 0.03). The heterogeneity between subgroups both conducted by race and ELISA kits was not significant with sharing the same studies of subgroup analysis (both I^2^ = 0%, *p* = 0.67).

There was a source of heterogeneity when stratified by sample size (I^2^ = 89.7%, *p* = 0.002). The irisin levels of the depressive patients were lower than those of non-depression with a sample size of more than 100 participants (SMD = 1.20, 95% CI: 0.48 to 1.93, *p* = 0.001), while there was no significant difference in those of less than 100 participants.

Additionally, other sources of heterogeneity were blood sample (serum versus plasma, I^2^ = 80.0%, *p* = 0.03) and gender (female only versus two genders, I^2^ = 86.9%, *p* = 0.006). Decreased circulating irisin levels were detected in depressive groups with subgroups using serum as blood sample (SMD = 0.79, 95% CI: 0.18 to 1.40, *p* = 0.01) and studies with two genders (SMD = 0.85, 95% CI: 0.26 to 1.43, *p* = 0.005). However, there was a subgroup with only two articles in each subgroup analysis conducted by the three factors above, which might affect the results because of the restricted scale of the study.

### 3.5. Second outcome

**Figure 3.**
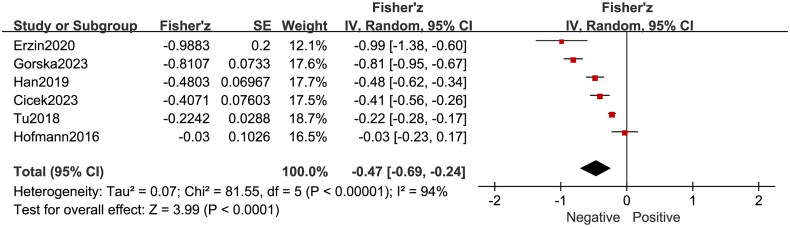
Forest Plot of meta-analysis with six studies on correlation between irisin levels and depressive scores.

Six studies [13,14,21–24] included in our meta-analysis reported negative correlation between irisin levels and depressive scores. The pooled correlation coefficient (r) also indicated the negative relationship (r = −0.47, 95% CI: −0.69 to −0.24, *p* < 0.001) (Figure 3).

### 3.6. Sensitivity analysis

Sensitivity analysis was conducted by the stepwise regression technique (Appendix 2). After excluding three of the included studies, respectively [13,14,22], the difference in circulating irisin levels between patients with depression and those without depression became statistically insignificant. This has affected the stability of the results, suggesting that there is a need to expand the sample size for future research.

### 3.7. Publication bias

The result of Egger’s test indicated that the publication bias was not significant in terms of irisin levels in cases compared with controls (*z* = 0.98, *p* > 0.05). However, with the limited study size, the power of Egger’s test was relatively low.

## 4. Discussion

As far as we know, this systematic review and meta-analysis is the first one that focuses on irisin with depression. This study revealed that the irisin levels in depressive individuals were lower than those in non-depressive individuals. Correlation analysis showed a negative correlation between circulating irisin levels and the depressive scores, indicating that the lower the circulating irisin level, the more severe the depressive symptoms. Our research findings are consistent with some of the literature. However, a few studies have shown that there is no difference in irisin concentration between healthy individuals and patients with depression [20,24]. The reason might be the differences in glucose and lipid metabolism among the subjects, which have been proven to affect irisin levels [25]. This suggests that these confounding factors need to be controlled in future studies.

Irisin reaches the brain through two common ways. Firstly, irisin in the peripheral circulation produced by muscle tissue after exercise can diffuse the brain, affecting the onset of depression. A study conducted on athletes and ordinary human subjects has found that irisin levels are negatively correlated with mild depression, supporting the above statement [26]. Kim et al. discovered higher irisin level in mice’s hippocampal tissue after intraperitoneal injection of irisin, confirming the existence of a transport pathway from periphery to the central nervous system [27]. On the other hand, irisin is synthesized by the brain, such as the midbrain and hippocampus. Cell studies have shown that during the maturation of cortical neurons in primary mouse embryos, the level of FNDC5, a precursor of irisin, increases [28]. The low expression of irisin in the brain led to a series of neurological and psychiatric disorders. Studies have found the decline of FNDC5 level in the prefrontal cortex in a mouse model of severe depression, while long-term administration of fluoxetine can increase the expression of the FNDC5 gene [29]. Because of the interconnection of irisin between inside and outside the brain, the level of peripheral irisin decreases along with the central one, which is proved by clinical studies [30]. Therefore, circulating irisin level can be a predictor of central disorders, which is meaningful for the diagnosis and treatment of these diseases.

There are currently two main hypotheses regarding the mechanism of irisin and depression. The first hypothesis is related to neurotrophins. It’s believed that a lack of neurotrophins will lead to neuronal atrophy, reduced neurogenesis, and destruction of glial cells, resulting in depression [31]. Brain-derived neurotrophic factor (BDNF) is one of the crucial neurotrophins. It is extensively expressed in the nervous system and has the ability to stimulate neurogenesis and modulate neuroplasticity, thereby improving brain functions such as emotion, cognition, and movement [32]. In the clinical application, some antidepressant drugs are associated with activating the BDNF system. More and more studies have made conclusions that irisin plays an important role in antidepressant effect by upregulating the level of BDNF in the brain [33]. Babaei et al. have found that after continuous and intermittent exercise, the levels of proteins with PGC-1α/FNDC5/irisin/BDNF pathway in the hippocampus of rats exposed to chronic unpredictable mild stress increase and the immobility time of forced swimming test decreases, demonstrating that the depressive symptoms are improved [34]. Besides, peripheral injection of irisin will significantly induce the expression of BDNF in the hippocampus and prefrontal cortex of mice, implying a positive effect on depression [35]. What’s more, irisin can also activate transient extracellular regulated protein kinases (ERK) phosphorylation, thus increasing the extracellular BDNF level, and protecting neurological function [36]. In our meta-analysis, three articles [15,21,24] described the levels of BDNF in cerebrospinal fluid or blood, suggesting a decrease in patients with depression, while two studies [15,21] found that irisin levels and BDNF levels were positively associated, which was in line with current research hypotheses.

Another hypothesis is about the relationship between irisin and neuroinflammation. It’s confirmed that irisin activates M2 macrophage polarization with the effect of peroxisome proliferator-activated receptor gamma (PPAR γ), thus reducing the level of inflammatory factors, like IL-1β, TNF-α and IL-6, and improving depressive symptoms induced by inflammation [37]. Moreover, irisin can inhibit inflammation by down-regulating the NF-kappaB pathway [38]. Recent research has demonstrated that there is an imbalance in mitochondrial homeostasis in the frontal lobe with depression [39]. As an important site for emotional development, the frontal lobe is related to the onset of depressive symptoms. The pathogenesis of mitochondrial homeostasis imbalance includes inflammatory infiltration, neuronal apoptosis, oxidative stress, and so on, leading to an increase in monoamine oxidase levels and a decrease in dopamine, resulting in depression [40]. Irisin can alleviate mitochondrial dysfunction by increasing the production of adenosine triphosphate (ATP), inhibiting mitochondrial oxidative stress and cell apoptosis, and stabilizing mitochondrial function while regulating metabolic disorders [41]. Furthermore, the latest research has shown that aerobic exercise in post-stroke depression model mice is able to inhibit the initiation of NF-kappaB/NLRP3 inflammasome through irisin and alleviate mitochondrial damage under stress by reducing calcium overload, thereby participating in the regulation of depression symptoms through the neurotransmitter of serotonin [42].

According to a previous study, a small sample size may lead to unreliable results [43]. Our subgroup analysis also suggested that sample quantity was a source of heterogeneity. In the subgroup with a large sample size, irisin levels were lower in patients with depression and negatively correlated with depressive scores, while the difference was not statistically significant in small sample size.

A meta-analysis conducted by X.L. Du corroborated that the combined results were not affected by the type of blood sample, while another study suggested the opposite conclusion [44,45]. In our subgroup analysis, the results were different between the subgroups of plasma and serum. However, with only two studies in subgroup of plasma, the results might be affected due to the small sample size.

What’s more, some researchers have reported that irisin level is directly linked to fat mass [46]. Therefore, the concentration of irisin may be higher in female than male, considering that women have higher body fat mass. Our subgroup analysis demonstrated the reduction of irisin levels in depression of studies with both genders, while no difference was found in studies with only females, indicating that further exploration should be conducted about irisin levels in different genders.

Furthermore, irisin level may vary in different ethnicities according to previous studies, although the mechanism is still unknown [47,48]. In our subgroup analysis, studies with non-Asians used ELISA kits from Phoenix Pharmaceuticals, while studies with Asians used ELISA kits from other companies. Therefore, the results of subgroup analysis stratified by race and ELISA kits were the same and probably influenced by the intersection of both factors. Besides, our meta-analysis showed that the measurement range of ELISA kits from different companies varied obviously, possibly due to the influence of operators or the quality of antibody, resulting in significant differences.

The results of subgroup analysis according to whether the subjects had other diseases showed that there were no differences in irisin levels between depressive and non-depressive patients within each subgroup. Although the depression and control groups shared the same disease in each study, ensuring comparability of results, more and more studies have found that some diseases are related to irisin, like type 2 diabetes, cognitive dysfunction and coronary heart disease, affecting the reliability of the results [11,49,50]. It is believed that the concentration of circulating irisin in patients with type 2 diabetes is decreased. Consistently, studies have found lower irisin levels in patients with cognitive impairment and coronary heart disease. Therefore, more subjects without comorbidities are needed to expand the study in the future.

Our research had multiple strengths. Previously, no systematic review had been conducted regarding the relationship between irisin and depression. Our meta-analysis not only compared the differences of the irisin levels between individuals with depression and non-depression, but also analyzed the correlation coefficient, giving new insight into the future research. Besides, the NOS scores of the studies included were all above five points, indicating high system quality.

In addition, our research findings have great clinical significance. Currently, there are no definitive clinical guidelines on the use of irisin levels in the diagnosis or monitoring of depression. Our research affirms the potential role of irisin as a biomarker, particularly in the diagnosis, treatment, clinical course and prognosis of depression. In the context of depression diagnosis, irisin has the likelihood of serving as a supplementary diagnostic indicator. By accurately measuring the irisin levels in patients and combining with other diagnostic methodologies, there is a promising prospect of enhancing the accuracy and specificity of the diagnostic process. During the treatment phase, the dynamic fluctuations in irisin levels can offer valuable insights for evaluating the treatment efficacy. Specifically, if the treatment is effective, a corresponding positive change in irisin levels may be observed. This information is crucial as it enables physicians to make timely adjustments to the treatment plan, optimizing the therapeutic approach. Moreover, irisin can be one of the indicators for prognosis. For instance, whether lower irisin levels indicate a higher likelihood of disease exacerbation or recurrence warrants further study. Additionally, if the irisin level gradually returns to normal during the process of treatment, it may suggest a better prognosis.

However, some limitations should not be ignored. As irisin is newfound, there are relatively few studies on irisin levels in relation to depression. Only eight qualified articles were incorporated in our meta-analysis, thus affecting the strength of the conclusions drawn from it. Secondly, due to the limited study size, populations with other diseases were not excluded, which increased the heterogeneity of the results. Current research has confirmed that BMI has an impact on irisin level with a positive correlation between them [51]. Regrettably, due to the lack of available BMI data in the original studies, our study couldn’t conduct a subgroup analysis by BMI. Besides, irisin in human body is mainly released by skeletal muscle after exercise, so that the state of exercise of each subject has a significant impact on the level of irisin. In a study on the serum irisin levels in patients with depression and the control group, subjects with heavy physical activities from both groups were excluded to draw a more reliable conclusion [25]. Nevertheless, this factor has not been considered in all studies included in our research.

Furthermore, the heterogeneity of depression is also a key consideration. Alterations in mental activities, fluctuations in appetite, and variations in sleep duration can exert a substantial impact on irisin levels. Nevertheless, as none of the included studies classified and compared irisin levels based on these symptoms, our meta-analysis was unable to conduct subgroup analyses in these aspects. Additionally, the differences in irisin levels between unipolar and bipolar depression, as well as its correlation with the depressive episodes and disease duration, are aspects worthy of exploration. However, among the included studies, only one conducted comparison in these aspects [24]. Its findings showed that irisin levels did not vary significantly between unipolar and bipolar depression, and in unipolar depressive patients, the irisin level was negatively correlated with disease duration and depressive episode, which confirmed that the irisin level was related to the severity of depression. Despite our inability to conduct subgroup analyses in these areas, this offers valuable insights for future research. Another factor that demands consideration is the link between irisin levels and circadian rhythm. While most of the included studies specified that sampling occurred in the morning following an overnight fast, two studies failed to show the sampling time. Hence, standardizing the sampling time is essential for future research.

## 5. Conclusion

In summary, this study revealed that peripheral irisin levels were significantly reduced in depressive patients and negatively correlated with depressive scores, confirming the role of irisin in depression. In the future, large-scale epidemiological investigations and research should be conducted, with a focus on controlling various confounding factors. Additionally, we call for more prospective cohort studies and randomized controlled trials, in order to further explore the diagnostic and therapeutic effects of irisin on depression.

## Supplementary Material

PRISMA0228.docx

Appendix.docx

## Data Availability

The data used in the current study are not publicly available because of the security but are available from the corresponding author upon reasonable request.
