# Supplementary material for: Association between circulating irisin level and depression: a systematic review and meta-analysis
Source: Ann Med. 2025 Jun 20;57(1):2521424. doi: 10.1080/07853890.2025.2521424 (PMC12931329; doi:10.1080/07853890.2025.2521424)
Supplement: Appendix.docx [file IANN_A_2521424_SM2291.docx]

**Appendix 1. Search Strategy**

| **Databases** | **Search terms** | **Numbers**  **of records** |
| --- | --- | --- |
| **Pubmed** | ((irisin[Title/Abstract] OR fndc5[Title/Abstract] OR "fibronectin type III domain-containing protein 5"[Title/Abstract]) OR ("FNDC5 protein, human" [Supplementary Concept])) AND ((depress*[Title/Abstract] OR dysthymi*[Title/Abstract] OR mood[Title/Abstract] OR affective[Title/Abstract] OR adjustment[Title/Abstract]) OR ((((("Depression"[Mesh] OR "Depressive Disorder"[Mesh]) OR "Mood Disorders"[Mesh]) OR "Adjustment Disorders"[Mesh]) OR "Affective Symptoms"[Mesh]) OR "Dysthymic Disorder"[Mesh])) | 120 |
| **Cochrane Library** | #1 MeSH descriptor:[Depression] explode all trees  #2 MeSH descriptor:[Depressive Disorder] explode all trees  #3 MeSH descriptor:[Mood Disorders] explode all trees  #4 MeSH descriptor:[Adjustment Disorders] explode all trees  #5 MeSH descriptor:[Affective Symptoms] explode all trees  #6 MeSH descriptor:[Dysthymic Disorder] explode all trees  #7 #1 OR #2 OR #3 OR #4 OR #5 OR #6  #8 (depress* or dysthymi* or mood or affective or adjustment):ti,ab,kw  #9 #7 OR #8  #10 (irisin or FNDC5 or “fibronectin type III domain containing protein 5”):ti,ab,kw  #11 #9 AND #10 | 17 |
| **EMBASE** | \| #1 \| 'depression'/exp \| \| --- \| --- \| \| #2 \| 'depression'/exp OR 'mood disorder'/exp \| \| #3 \| 'depression'/exp OR 'adjustment disorder'/exp OR 'mood disorder'/exp \| \| #4 \| 'depression'/exp OR 'adjustment disorder'/exp OR 'mood disorder'/exp OR 'emotional disorder'/exp \| \| #5 \| 'depression'/exp OR 'adjustment disorder'/exp OR 'mood disorder'/exp OR 'emotional disorder'/exp OR 'dysthymia'/exp \| \| #6 \| depressive OR 'depression'/exp OR depression OR 'depressivity'/exp OR depressivity OR affective OR 'mood'/exp OR mood OR dysthymic OR 'dysthymia'/exp OR dysthymia OR 'adjustment'/exp OR adjustment \| \| #7 \| depressive OR depression:ab,ti OR depressivity:ab,ti OR affective:ab,ti OR mood:ab,ti OR dysthymic:ab,ti OR dysthymia:ab,ti OR adjustment:ab,ti \| \| #8 \| #5 OR #7 \| \| #9 \| 'irisin'/exp \| \| #10 \| 'irisin'/exp OR irisin OR fndc5:ab,ti OR 'fibronectin type iii domain-containing protein 5':ab,ti \| \| #11 \| #8 AND #10 \| | 180 |
| **Web of Science** | (TS=(depress* or dysthymi* or mood or affective or adjustment or mood)) AND TS=(irisin or FNDC5 or "fibronectin type III domain-containing protein 5") | 144 |
| **Scopus** | TITLE-ABS-KEY ( "depress*"  OR  "dysthymi*"  OR  "mood " OR  "affective" OR  "adjustment") AND TITLE-ABS-KEY( "irisin"  OR  "fndc5"  OR  "fibronectin type III domain-containing protein 5" ) | 172 |

**Appendix 2.**

Table A1. Sensitivity analysis of circulation irisin levels in subjects with depression and non-depression

| Study of omission | Heterogeneity | | Meta-analysis | |
| --- | --- | --- | --- | --- |
|  | *I^2^* | *p* value | SMD（95%CI） | *p* value |
| Gonçalves 2022 | 95% | ＜0.001 | 0.68(0.12,1.24) | 0.02 |
| Cicek2023 | 95% | ＜0.001 | 0.51(-0.08，1.10) | 0.09 |
| Han2019 | 95% | ＜0.001 | 0.65(0.05，1.24) | 0.03 |
| Tu2018 | 95% | ＜0.001 | 0.56(-0.18，1.30) | 0.14 |
| Gorska2023 | 88% | ＜0.001 | 0.34(-0.03，0.72) | 0.08 |
| Samanci2019 | 95% | ＜0.001 | 0.73(0.19，1.27) | 0.008 |
| Hofmann2016 | 95% | ＜0.001 | 0.69(0.13，1.25) | 0.02 |
| Erzin2020 | 95% | ＜0.001 | 0.67(0.10，1.23) | 0.02 |
| All studies without omission | 95% | ＜0.001 | 0.60(0.08,1.12) | 0.02 |
